# Evidence-Based Intervention (EBI) Mapping: a systematic approach to understanding the components and logic of EBIs

**DOI:** 10.1186/s12889-022-13692-x

**Published:** 2022-07-07

**Authors:** Timothy J. Walker, Maya Foster, Jacob Szeszulski, Derek W. Craig, Patricia Dolan Mullen, Maria E. Fernández

**Affiliations:** 1 Department of Health Promotion & Behavioral Sciences, Center for Health Promotion and Prevention Research, University of Texas Health Science Center at Houston School of Public Health, Houston, 7000 Fannin St., TX 77030 USA; 2 Department of Health Promotion & Behavioral Sciences, Center for Health Promotion and Prevention Research, Michael & Susan Dell Center for Healthy Living, University of Texas Health Science Center at Houston School of Public Health, 7000 Fannin St., Houston, TX 77030 USA

**Keywords:** Program Planning, Intervention Mapping, Adaptation, Implementation

## Abstract

**Background:**

Despite the development of numerous evidence-based interventions (EBIs), many go unused in practice. Hesitations to use existing EBIs may be due to a lack of understanding about EBI components and what it would take to adapt it or implement it as designed. To improve the use of EBIs, program planners need to understand their goals, core components, and mechanisms of action. This paper presents EBI Mapping, a systematic approach based on Intervention Mapping, that can be used to understand and clearly describe EBIs, and help planners put them into practice.

**Methods:**

We describe EBI Mapping tasks and provide an example of the process. EBI Mapping uses principles from Intervention Mapping, a systematic framework for planning multilevel health promotion interventions. EBI Mapping applies the Intervention Mapping steps *retrospectively* to help planners understand an *existing* EBI (rather than plan a new one). We explain each EBI Mapping task and demonstrate the process using the VERB Summer Scorecard (VSS), a multi-level community-based intervention to improve youth physical activity.

**Results:**

EBI Mapping tasks are: 1) document EBI materials and activities, and their audiences, 2) identify the EBI goals, content, and mechanisms of action, 3) identify the theoretical change methods and practical applications of those methods, 4) describe design features and delivery channels, and 5) describe the implementers and their tasks, implementation strategies, and needed resources. By applying the EBI Mapping tasks, we created a logic model for the VSS intervention. The VSS logic model specifies the links between behavior change methods, practical applications, and determinants for both the at-risk population and environmental change agents. The logic model also links the respective determinants to the desired outcomes including the health behavior and environmental conditions to improve the health outcome in the at-risk population.

**Conclusions:**

EBI Mapping helps program planners understand the components and logic of an EBI. This information is important for selecting, adapting, and scaling-up EBIs. Accelerating and improving the use of existing EBIs can reduce the research-to-practice gap and improve population health.

## Background

Evidence-based interventions (EBIs) are broadly defined as programs, practices, processes, policies, and guidelines that have proven efficacy or effectiveness in a population and setting [1]. Researchers have produced numerous EBIs shown to improve health outcomes, yet many EBIs go unused in practice [2, 3]. This is, in part, because existing EBIs rarely fit seamlessly into a context or setting that is different from the one in which they were originally developed and tested [4]. Even when there is good potential fit, this is not always clear to those responsible for making decisions about whether to use an EBI. To complicate matters, many EBIs are multilevel (i.e., target more than one system level such as individuals, organizations, communities) and/or have multiple components (i.e. include various parts that work synergistically) [5]. The complexity of EBIs can cloud planners’ decisions about selection and the potential adaptation (i.e. changes to improve fit to local conditions) needed [1].

When selecting EBIs, program planners must consider multiple factors, including features of the intervention, the strength of the evidence, and potential fit with the new target population and setting [6]. Resources such as Evidence-Based Cancer Control Programs (formerly RTIPs) [7], National Registry of Evidence-based Programs and Practices [8] and others, provide access to EBIs including general descriptions of the EBI, evidence of effectiveness, populations of focus, and setting. While some resources highlight the EBI’s core elements (those essential components that make the program effective), many do not. Thus, when determining if an existing EBI *could* work in a new setting/population, the challenge of knowing what could change and what should stay the same persists. Thus, methods to better understand the core elements of EBIs are needed.

There are ongoing efforts to improve intervention reporting that include a focus on specifying not only the goals and various components of an intervention, but also its theory of change (causal mechanisms that link intervention methods and strategies to the determinants) [9, 10]. Nevertheless, few existing intervention reports clearly articulate this information, which leaves program planners in a position to decipher the goals, explicit targets, and mechanisms (or logic) of change of a given EBI. This difficulty also hampers adaptation efforts. Although existing adaptation frameworks can help guide program planners through the adaptation process, a critical step in the process is to clearly understand the EBI, which includes identifying the underlying theory and core elements [11, 12]. This information is critical for understanding *why* an intervention was effective and can guard against changing elements of the EBI that interfere with its “mechanisms of action” and thus, effectiveness. Henceforth, we call this the EBI’s “logic”.

To address the difficulty of determining an EBI’s logic, we propose “EBI Mapping”, a systematic approach to analyzing and describing an EBI. EBI Mapping is based on Intervention Mapping [13], which is a systematic protocol used to plan multi-level interventions that has been used globally in many populations and settings [14]. Although Intervention Mapping is traditionally used prospectively to develop interventions, its principles can be applied retrospectively to “reverse engineer” or “map” an existing EBI [15–17]. Intervention Mapping uses logic models to describe how the various methods and strategies in an intervention influence health. Logic models typically describe causal relations between determinants and outcomes, usually graphically, and they can be extremely useful for understanding an EBI [18, 19]. The purpose of this paper is to present the EBI Mapping process. The methods section includes a brief description of the development of EBI Mapping and how it can be used to better understand an existing intervention. The results section includes a detailed explanation of the EBI Mapping tasks and an example of EBI Mapping applied to a multi-level, community-based intervention to increase physical activity among youth.

## Methods

### Development of EBI Mapping tasks

EBI Mapping is based on Intervention Mapping [13], which provides terms, guiding questions, and attention to mechanisms of action (e.g., it explicitly links behavioral determinants to behavior change methods through a series of steps) for developing multilevel interventions. EBI Mapping is also based on IM-ADAPT, a process for adapting EBIs based on Intervention Mapping [15, 16]. A key component of IM-ADAPT, which was refined during the development of *IM-ADAPT Online* (an online tool to assist in EBI adaptation), is a process for “reverse-engineering” an existing EBI. This process, examining the materials and other information about an EBI to determine its goals, target audiences, proposed targets for behavioral and environmental change, and mechanisms of change for influencing determinants (intervention logic), is what we refer to as “EBI Mapping” [14, 16, 20–22]. As part of a National Cancer Institute-funded contract to better describe colorectal cancer screening interventions listed on the National Cancer Institute’s Evidence-Based Cancer Control Programs website, the team further validated and refined the EBI Mapping process [23]. Based on the experience of multiple coders and team discussions we developed resources (e.g., a workbook and online tool) to help users through the process.

**Fig. 1:**
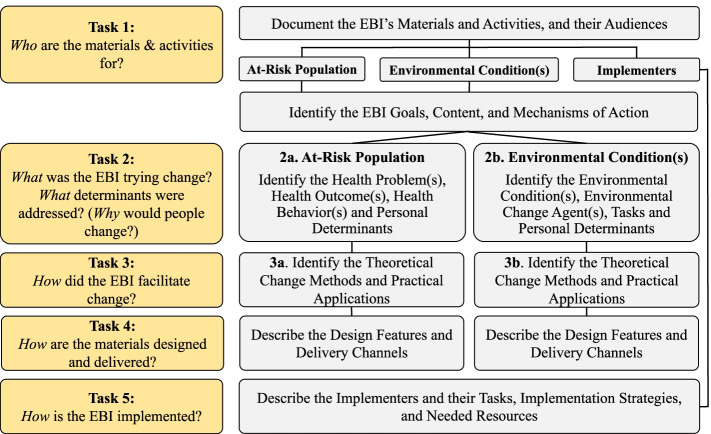
EBI Mapping Tasks

EBI Mapping tasks identify the *who* (i.e., intervention targets including the at-risk population and/or environmental conditions), *what* (i.e., health behavior and health problem), *why* (i.e., personal determinants), and *how* (i.e., EBI theoretical change methods and practical applications of those methods) of an EBI (Fig. 1). The five tasks also help planners document design characteristics, delivery channels, implementation strategies used, and the resources needed to deliver the intervention. The tasks are completed iteratively, meaning that although planners work through them systematically to map the components of an EBI, they often go back and forth between tasks sometimes identifying an element in a later task before an earlier task. At the end of the process, a planner will have created a complete logic model of the EBI and will have described the EBI’s design and delivery features.

### EBI Mapping

**Fig. 2:**
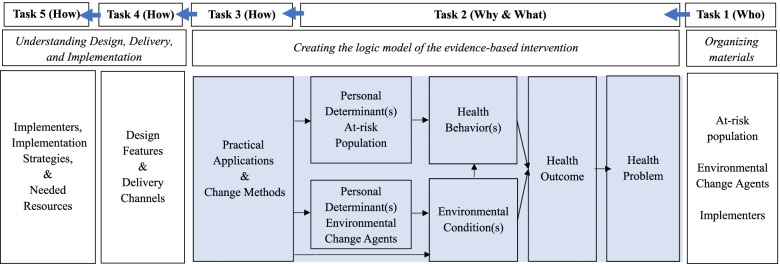
EBI Mapping Process

Figure 2 displays the EBI Mapping approach including each task and how it corresponds to the creation of the logic model of the EBI. Notably, Task 1 begins on the far-right side of the model where a user first organizes the materials and identifies who they are for. As a user completes Tasks 2&3, they create the logic model for the EBI by explicitly linking the health outcome to determinants and change methods. Thus, the tasks are completed working from right to left, while specifying the causal logic for how an EBI achieves outcomes, from left to right.

## Using EBI mapping to understand the Verb Summer Scorecard (VSS)

The Centers for Disease Control introduced the Verb Summer Scorecard (VSS) intervention [24, 25] in 2002 as a national campaign that uses mass media to promote physical activity among “tweens” (youth ages 9–13 years) [26]. The VSS intervention is a local extension of the national VERB campaign and focuses on promoting summer activities among youth to increase opportunities for physical activity. The intervention involves assembling a community coalition, recruiting local businesses and organizations to participate by supporting tween physical activity, and providing tweens with a scorecard to track their physical activity. We downloaded VSS materials from the Center for Training and Research Translation (Center TRT) website (centertrt.org), as well as published articles about the intervention to carry out the EBI Mapping process. Two team members (TW and MF) led the EBI Mapping process for the VSS intervention with additional support from the research team. The subsequent results section describes each EBI Mapping task in detail together with results from mapping the VSS intervention.

## Results

### Task 1: Document the EBI’s materials and activities and their audiences

At the start of Task 1, a planner needs to inventory the materials and activities included in the EBI and determine whom each of the materials and activities are designed for. Materials should be organized by audience type, which include (1) *at-risk population* (those who have the health problem or are at risk of acquiring a health problem as the result of a behavior or environmental exposure), (2) *environmental change agents* (those who can bring about change in the at-risk population’s interpersonal, organizational, community, or policy environment), and/or (3) *implementers* (those responsible for delivering the EBI). Identifying the audience helps to determine which groups the EBI targets, because some EBIs target only the at-risk population directly, others target *environmental conditions* only, and some may target both (e.g., multilevel interventions). In Task 1, a program planner also needs to further describe the at-risk population and setting of the EBI as well as link specific intervention materials to the respective audience type. Definitions and examples of each term are presented in Table 1.

**Table 1:** Intervention Mapping Terms

Construct	Definition	Examples
Health outcomes	Prevention, reduction, or control of the health problem	Early detection of breast or colorectal cancer, prevention of Hepatitis B or HPV infection, or a reduction in weight/adiposity
Health problems	A deficit of health, excess of disease or risk factor for disease in a defined population	Breast cancer, colorectal cancer, Hepatitis B infection, HPV infection, obesity
Health behaviors	An action or set of actions performed by the population of interest that is expected to decrease the health problem or decrease complications or increase quality of life	Cancer screening test, physical activity, healthy eating, smoking cessation, HPV vaccination
Sub-behaviors	The specific actions targeted that contribute to a broader health behavior	Scheduling a visit for a mammogram, limiting sugar sweetened beverages
Personal determinants	Factors that reside within an individual that influence their behaviors	Knowledge, attitudes, or outcome expectations
Environmental conditions	Factors in an individual’s social or physical environment (surroundings) that influence the health of the at-risk population or their behaviors	See examples below
Interpersonal	Individuals in the person’s immediate environment (e.g., parents, other family members, peers) who have influence on the at-risk population	Family member who provides social support Medical provider who gives a referral for a cancer screening
Organizational	Aspects of characteristics of organizations that influence the behavior of the at-risk population	Worksite smoking ban. Private rooms available to nursing mothers for breastfeeding or pumping
Community	Aspects of a community that influence the behaviors or affect the health of the at-risk population	Restrictions on where people can smoke to avoid secondhand smoke exposure
Societal	Policies, facilities, and interventions of larger political and geographic locations that affect the health and behavior of the at-risk population	Legislation that limits tobacco sales to minors
Change methods	Techniques or processes for influencing positive change in the determinants of behaviors	Discussion, modeling, and/or tailoring
Practical Applications	A specific technique for the practical use of a theoretical change method used by an EBI	Using images of people doing the health behavior on print materials as a form of modeling

#### Task 1: Documenting VSS materials, activities, and audiences

Table 2 lists the 14 VSS intervention materials (and their corresponding audiences) provided by the Center TRT website. VSS intervention developers clearly defined the at-risk population as “tweens” in the United States. We identified materials for business and facility managers, whom we determined to be environmental change agents, and for coalition members, whom we determined to be the implementers. We did not include evaluation surveys and interview guides that were also provided on the Center TRT website because they were not part of the intervention. Also, we did not include a five minute promotional video played in schools and endorsements in school newsletters that were mentioned in a published article because these materials [25] were not accessible on the Center TRT website.

**Table 2:** Task 1 Documenting VERB Summer Scorecard Materials and Audience

Material	Audience
1. 2004 Scorecard	At-Risk Population (“Tweens”, or youth ages 9–13 years)
2. 2005 Scorecard	
3. Tween incentives	
4. Vendor Recruitment Letter	Environmental Change Agents (Business/facility managers)
5. VERB Logo-use Terms and Conditions	
6. Designing a Successful VERB Scorecard Campaign in Your Community	Implementers (Coalition Members)
7. Marketing Plan Concepts and Questions to Consider	
8. Promoting Physical Activity in Community Settings: A Strategy Formation Workbook	
9. Keep it Fun	
10. Move Kids to Action	
11. Having a Successful Physical Activity Event: Your Guide to Making Physical Activity Appealing to 9- to 13-Year-Olds	
12. Event Logistics Guide: Planning a Community-Wide VERB Activity Zone Event	
13. VERB Logo Files	
14. Vendor Monitoring Forms (Six Word and PDF Versions)	

### Task 2: Identify the EBI goals, content, and mechanisms of action

Task 2 begins the development of a logic model by identifying the key contents of the EBI (primary outcomes and factors addressed to impact the outcomes). For EBIs that target the at-risk population, a planner needs to identify the *health problem(s)*, *health outcome(s), health behavior(s)*, and *personal determinants*. For EBIs that target environmental conditions that influence the behavior of the person in the at-risk group or influence health directly, a planner needs to identify the level (interpersonal, organizational, community, etc.), the *environmental change agent(s)*, the change agent’s tasks (or behaviors), and personal determinants influencing change agent actions. For EBIs that address only environmental conditions (and not the at-risk population’s behavior directly), it is important to identify the health problem, health outcomes, and any behaviors (of the at-risk population) that are intended to be affected by the targeted change in environment. It is not necessary, however, to list personal determinants influencing behavior for the at-risk population, because the intervention did not include materials or protocols designed for them. Similarly, some EBIs may not address environmental conditions, and, thus, a planner can just focus on the at-risk population.

### Task 2 a: Identify the health problem(s), health outcome(s), health behavior(s), and personal determinants for the at-risk population

A planner should first determine the health problem(s) that the EBI addresses and the health outcome(s) expected. The health outcome is the type of change the EBI is trying to make in the health problem (e.g., prevent, reduce, control). Next, the planner describes the health behaviors (and sub-behaviors) the EBI is expected to influence to achieve the health outcome. Sub-behaviors (called performance objectives in Intervention Mapping) are the specific actions that must be completed to accomplish the broader health behavior. For example, an EBI targeting mammography screening might address sub-behaviors including: asking the doctor for a mammography referral, scheduling the appointment, getting screened, and getting results [27]. A planner should list only the health behaviors, health problems, and health outcomes that the EBI addresses explicitly. If the EBI addresses more than one health behavior, outcome, or problem, then each should be listed.

The planner should then identify the determinants (factors influencing) of the positive health behavior targeted by the EBI. Usually, EBIs aim to influence personal determinants related to the health behavior (and sub-behaviors) of the at-risk population. These factors are typically constructs from behavioral theories or frameworks, such as *knowledge*, *attitudes*, and *outcome expectations.*

#### Task 2 a: Identifying the VSS health problem, health outcome, behaviors, sub-behaviors, and personal determinants

The health problem addressed by VSS, overweight and obesity, was described throughout the materials (Table 3). Based on the health problem, we inferred the desired health outcome as prevention and reduction of overweight and obesity. Materials for all target audiences indicate that the desired health behavior for tweens is increasing physical activity. From the materials for tweens (VSS scorecards), we identified the sub-behavior as the tweens selecting activities to engage in. We also used the VSS scorecards to identify personal determinants, which included: overcoming barriers, attitudes, outcome expectations, social norms, and knowledge. Table 3 includes the determinants and how we identified them on the VSS scorecards.

**Table 3:** Task 2a—Health Problem, Health Outcome, Behaviors, Sub-behaviors, and Personal Determinants

Construct	VSS Example
Health problem	Overweight and obesity
Health outcomes	Prevention of overweight and obesity
Health behavior	Physical activity Tweens select activities to engage in
Sub-behaviors	
Personal determinants	*Overcoming barriers*—community activities listed on scorecard *Attitudes* & *Outcome Expectations—*emphasis on physical activity as “fun” and enjoyable on scorecard *Social Norms*—scorecards include pictures of tweens *Knowledge*—scorecards list ways and paces to be active

### Task 2 b: Identify the environmental condition(s), environmental change agent(s), environmental change tasks, and determinants

Environmental conditions can occur at different social-ecological levels and affect health directly (e.g., air pollution) or influence the behaviors of individuals through contextual factors (e.g., access to services, transportation, and provider recommendation). To identify whether an EBI targeted environmental conditions, a program planner needs to determine what changes the EBI targeted in the at-risk population’s environment that would improve their health outcomes or health behaviors. Planners also need to determine the level at which the EBI has targeted changes in the environmental condition (*interpersonal*, *organizational*, *community*, or *societal*).

To change an environmental condition, EBIs can include methods and strategies that are designed to influence the environmental condition directly (e.g., forming coalitions to create a more supportive health environment), or methods and strategies to influence someone (or group) who has the power or opportunity to make an environmental change. That person is referred to as an environmental change agent (or environmental actor). Sometimes, environmental change agents can be individuals who have influence over the at-risk population’s behavior (e.g., a teacher or provider), or, who can modify an important condition in the environment (e.g., clinic hours, transportation) that can influence the at-risk population’s health either directly or by influencing the person’s health behavior. Change agents can be a single type of person (e.g., medical care providers, parents), a group (e.g., city council, school board), or several types of people (e.g., teachers and principals). After identifying change agents, a planner then determines the specific tasks that were targeted by the EBI (e.g., determine the need for vaccination and provide a recommendation) that the environmental change agent should do to change the environmental condition. The environmental change agent’s ability to carry out a task is influenced by determinants. Thus, a planner also needs to identify which personal determinants the EBI addresses to influence the tasks (behaviors) of an environmental change agent.

#### Task 2 b: Identifying VSS environmental conditions, environmental change agents, tasks (behaviors), and personal determinants

The environmental conditions that VSS seeks to change are increasing physical activity opportunities for tweens (community level) and increasing parental support (interpersonal level) (Table 4). We identified this information from the physical activity event planning guide, marketing strategy handbook, and scorecards. We also noted that the EBI targeted environmental change agents including businesses/facilities and staff, and parents. Specific tasks and determinants for environmental change agents are provided in Table 4. The partner recruitment letter highlights the benefits of physical activity for children and the benefits of business/facility participation in VSS to influence a business leader’s personal determinants of *knowledge*, *attitudes*, and *outcome expectations*. Even though there were no materials created directly for parents, the scorecards included a place for parents to stamp/sign when their tween completed an hour of physical activity. Parents are also likely to see information about physical activity opportunities and discounts on the scorecards, which may indirectly influence their *knowledge* about physical activity opportunities and *overcoming barriers* to support their child’s participation.

**Table 4:** Task 2b—Environmental Conditions, Change Agents, and Tasks

Environmental Condition (Level)	Environmental Change Agent	Task	Personal Determinants
Increasing physical activity opportunities for tweens (Community)	Businesses/facilities leaders and staff	• Agree to participate/complete registration form • Identify or select ways to partner • Stamp or sign tween scorecards	Knowledge Attitudes Outcome Expectations
Increasing parental support for tween physical activity (Interpersonal)	Parents	• Sign or initial tween scorecards • Transport tweens to activities	Knowledge Overcoming Barriers

### Task 3: Identify the theoretical change methods and practical applications for the at-risk population and environmental conditions

In addition to identifying determinants, a planner needs to identify *theoretical change methods* (general techniques for influencing change in the determinants of health behaviors) and *practical applications* (a specific technique for the practical use of a change method) that the intervention used for both the at-risk population and environmental conditions. The practical applications need to be linked to the change methods, and the change methods need to be linked to the personal determinants. The change methods (within practical applications or strategies) and the determinants they are intended to influence represent the mechanism through which the EBI is designed to influence outcomes. An example change method is *modeling* and a corresponding practical application is *video-recorded role models* (a more complete list is available as part of the EBI Mapping materials). Sometimes, practical applications and/or change methods are more identifiable in EBI materials than determinants. Thus, a planner can examine the practical applications (materials and activities used in the EBI), identify what theoretical change methods they contain, and infer what personal determinants were being addressed [28].

To target environmental conditions, some EBIs use change methods that do not operate through personal determinants of an environmental change agent. Instead, these change methods directly target the environment, a process, and/or a system that can affect the at-risk population’s health behavior (i.e., environmental change methods). A planner should note these methods because they are important components of how the EBI addresses the at-risk population’s environment and, ultimately, their health.

#### Task 3: Identifying VSS theoretical change methods and practical applications for the at-risk population and environmental conditions

**Fig. 3:**
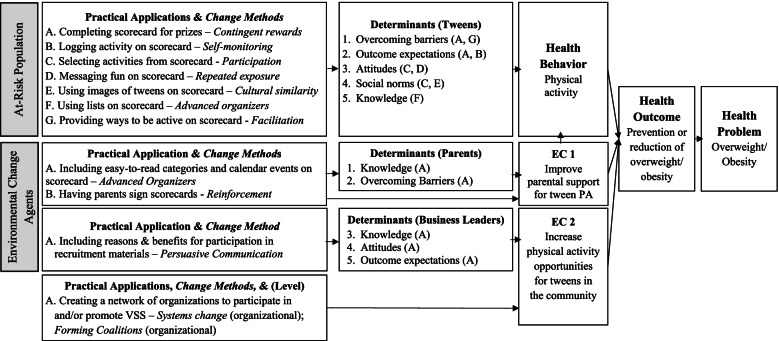
Logic Model of VERB Summer Scorecard Intervention (EC, Environmental Conditions)

We closely examined the scorecards to understand how they were being used to affect changes in determinants of tween and parent behavior. We sought to identify the change methods that scorecards operationalized (Table 5). We used the vendor recruitment letter to identify the change methods for business leaders. We also noted that the VSS intervention targeted the community environment directly (not through a change agent) by using *systems change* and *forming coalitions* (change methods) by *creating a network of organizations to participate in and/or promote VSS* (practical application). We then linked change methods to personal determinants, using EBI Mapping resources, which include tables of change methods and commonly targeted determinants and applications. By linking determinants, change methods, and practical applications, we created the logic model of the VSS intervention (Fig. 3). The logic model provides descriptive information (health problem, at-risk population, setting, environmental change agents) and a causal path for how the intervention components (practical applications and change method) connect to positively influence the determinants, health behavior, environmental conditions, and health outcomes. The logic model also highlights the function (i.e., the change processes of the EBI – behavior change methods and determinants), and the form (i.e. the specific ways to carry out the function – practical applications) of the EBI [29].

**Table 5:** Task 3 – Identify change methods & practical applications

Audience	Change Method	Practical Application
Tweens	Contingent rewards	Completing scorecards for prizes
	Self-monitoring	Logging activity on scorecards
	Participation	Selecting activity on scorecard
	Repeated Exposure	Multiple positive messages on scorecard
	Cultural Similarity	Using images of tweens on scorecard
	Advanced Organizers	Using lists on scorecard
	Facilitation	Providing ways to be active on scorecards
Parents	Advanced Organizers	Using lists on scorecard
	Reinforcement	Having parents sign scorecards
Business Leaders	Persuasive Communication	Including benefits for participation in recruitment materials
Community Environment	Systems Change	Creating a network of organizations to participate and/or promote VSS
	Forming Coalitions

### Task 4. Describe the design features and delivery channels for the at-risk population and environmental change agents

Task 4 consists of identifying design features and delivery channels for the EBI. Design features are important to understanding the visual and communication elements of the EBI and include: *look and feel* (appearance and impression, colors, layout, font, menus, buttons, pictures, page length of materials, and the duration of activity), *cultural elements* (symbols, quotations, colors, scenes, or any other elements intended to depict shared values and norms of a particular group or subgroup), *language* (written or spoken), *readability* (the ease with which a reader can understand a written text, including typographic aspects, such as the type, size, and spacing of the font), *data and statistics* (mode of presentation and whom the data are about), and *branding and contact information* (information specific to the settings that previously delivered the EBI, e.g., logos, phone numbers, website information). A planner can describe design features for a set of materials for a specific audience (e.g., a series of health promotion posters for the at-risk population) or for each material, depending on the characteristics of the EBI.

Delivery channels are the means through which an EBI is delivered (who delivers the material/activity, how often, when, and how, e.g., text, TV monitors, billboards) and provide important logistical information when considering use of an EBI. Delivery channels are especially important for a planner to consider when thinking about adaptation, implementation, and the resources required. For example, an EBI that uses a video as a delivery channel may be more difficult to adapt for a different at-risk population compared to an information sheet.

#### Task 4: Describing VSS design features and delivery channels

The materials for the at-risk population include the two different versions of the VSS scorecards while the materials for the environmental change agents include a recruitment letter and the terms and conditions for using the VERB logo (Table 6). The scorecards are intended to be delivered widely throughout the community in print and PDF forms (small media) as are the vendor recruitment letter and logo-use document. Suggested ways to deliver the scorecards include partner/vendor locations, schools, afterschool programs, youth clubs, and other businesses frequented by tweens (e.g. restaurants, movie theaters). The original VSS intervention delivered materials through schools by having physical education teachers distribute scorecards to students; principals promote the intervention using videos, morning announcements, and newsletters; and the school district superintendent feature the VSS intervention on the district website [25].

**Table 6:** Task 4 – Describe the design features & delivery channels

**Design Feature**	**VSS Scorecards** (At-Risk Population)	**Recruitment Letter (RL); Terms & Conditions form (TC)** (Environmental Change Agents)
Look & Feel	Bright, multicolored scheme, includes front/back	4-page document, black & white text (RL); 6-page document, black & white text and blue subheadings (TC)
Cultural Elements	Photos of racially/ethnically diverse tweens	No cultural elements
Language	English	English
Readability	8^th^ grade reading level	9^th^ grade reading level (RL); 10^th^ grade reading level (TC)
Data and statistics	No data/statistics	About brand awareness of VERB national campaign (RL)
Branding & contact information	VSS logo and website	Information about dates, locations, and contact information as placeholders (RL); fax number and address of national VERB Campaign Partnership Team (TC)

### Task 5: Describe the implementers and their tasks, implementation strategies, and needed resources

Finally, Task 5 focuses on describing intervention implementers, their tasks delivering the EBI, implementation strategies, and resources necessary to implement the EBI. Implementers are individuals or groups responsible for delivering each EBI material and activity to the at-risk population and/or environmental change agent or those individuals or groups enabling implementation to happen. This can include leaders or coordinators who create a supportive environment for implementation or who make changes in contextual factors [30, 31]. Some EBIs will have implementation manuals that specify tasks for the implementers to ensure implementation with fidelity. Implementation strategies are methods or techniques used to enhance the adoption, implementation, and sustainability of an EBI [32]. Listing the implementers, tasks, and strategies helps to identify and organize who is involved with EBI delivery and the ways to ensure effective implementation of the intervention (e.g., training). Resources needed to deliver the EBI also are important to review and document. Resources include money, staff, time, equipment, and so forth.

#### Task 5: Describing VSS implementers and their tasks, implementation strategies, and needed resources

Coalitions are the primary implementers tasked with developing infrastructure for VSS delivery. The “Designing a Successful VERB Scorecard Campaign in Your Community” document describes detailed implementer tasks and implementation strategies including (1) identifying a lead agency, (2) establishing a planning group and subcommittees, (3) recruiting local businesses and non-profit agencies to become partners, (4) designing and testing the scorecard, (5) defining campaign logistics (e.g. identifying target segment of tween population, behavioral goal, time frame, participation requirements, prizes and incentives), (6) developing a marketing plan, and (7) planning and hosting community-wide physical activity events. The “Event Logistics Guide” describes tasks and implementation strategies associated with the community events as (1) selecting event locations, (2) selecting appropriate “fun” activities and interactions for tweens, (3) recruiting quality staff to work the events, (4) training event staff, (5) establishing policies for event staff (e.g., behaviors, responsibilities, attire/appearance), (6) developing talking points for staff, (7) ensuring that the appropriate equipment is available (e.g., sidewalk chalk, cones, balls), and (8) selecting event prizes and incentives.

As indicated in the “Designing a Successful Scorecard Campaign” document, the cost of implementing the VSS can range from $100 to $35,000 depending on the scope and scale of the campaign. The Center TRT website describes staffing needs for complete implementation. Time is also needed for coalition members to recruit business and facilities and to monitor participating vendors, plan community events, develop marketing plans, and marketing training. The “Designing A Successful VERB Scorecard Campaign” document suggests at least 6-month planning period, with 9–12 months being preferable. Necessary equipment could also include spaces for coalition meetings, planning sessions, and community events.

## Discussion

EBI Mapping provides a step-by-step process for unpacking and clearly describing the components and logic of an EBI. We presented the process, explained the tasks, and provided an example using the VSS intervention. We specified personal determinants for the at-risk population and environmental change agents (business/facility, staff, and parents) as well as change methods and practical applications for changing these determinants and environmental conditions. With this information a program planner is better positioned to make decisions about whether an EBI is optimal for their intended population and is better informed about adaptation needs.

EBI Mapping helps identify intervention components over and above more superficial features typically available on websites or published articles such as population, behavior, setting, and delivery channels. By identifying determinants, change methods, and practical applications, a planner can better understand the form and function of an EBI and determine potential intervention fit and adaptation needs [33]. For example, if a program planner were interested in using the VSS intervention with a different population (e.g., teens ages 14–18 years), methods that target barriers, such as including information about age-appropriate community activities, are likely effective and should be maintained. This is because overcoming barriers is a determinant of physical activity for adolescents (10–18 years) [34, 35]. In contrast, a planner might consider adapting other components that could be better aligned. For example, a planner may emphasize messaging to more explicitly target self-efficacy as there is consistent evidence that self-efficacy is a determinant of physical activity for teens [36].

EBI Mapping also helps improve understanding of design, delivery, and implementation of EBIs. This information can further inform adaptation decisions such as substituting age-appropriate images and activity opportunities if using the VSS intervention with a different at risk population. The materials for implementers and implementation strategies provide information about how to best implement the VSS intervention with success (e.g., training event staff). The EBI Mapping principles can be further applied to unpack how implementation strategies used within an EBI influence outcomes [30, 37]. Thus, a user could develop both a logic model for the EBI and the implementation strategies used to deliver the EBI. Further understanding implementation strategy mechanisms is important for EBI success and an area of future work [38, 39].

Having a better understanding of EBI components can improve their use across populations and settings to maximize their generalizability and benefit. Most reports of EBIs lack the details required to conduct EBI mapping. For example, current guidelines do not include determinants and theoretical change methods – essentially, the mechanisms through which interventions are expected to create change in outcomes [10]. Ongoing efforts to improve intervention reporting through improved reporting of determinants and theoretical change methods and required availability of intervention materials will help developers to specify these components [9, 28]. These steps are critical for future work to facilitate the replication of promising interventions and improve EBI use in practice.

We believe EBI Mapping is a promising method to improve our understanding of the underlying mechanisms through which EBIs operate and has many favorable features. EBI Mapping is based on a systematic intervention development approach, Intervention Mapping, which explicitly links determinants to behavior change methods. In addition, extensive work on the IM adaptation framework, particularly on IM-Adapt Online, has specified the need for and tasks of EBI Mapping, leading to newly developed resources to help program planners work through the specific tasks. The resources for EBI Mapping include step-by-step guidance to create a logic model of action, a glossary of terms to maintain consistent nomenclature throughout the process, examples for how to identify intervention components, and lists of commonly used determinants and behavior change methods.

### Limitations of the EBI Mapping process

A program planner needs to have all intervention materials to carry out the EBI Mapping process. Access to materials has greatly improved through the growing number of resources (e.g., evidence-based cancer control programs [7], National Registry of Evidence-based Programs and Practices [8]), although most sources do not guarantee access to materials. It can take time for effective programs to be incorporated into these resources, and intervention developers need to be willing to share their materials. There can also be inconsistencies between resources such as published journal articles and the intervention materials (e.g., determinants listed in a manuscript that are not identifiable in the materials). These inconsistencies can introduce challenges when specifying determinants, change methods, environmental change agents, and implementers [23].

EBI Mapping is a detailed process that sometimes requires a planner to make judgments about which determinants or methods are being used or which environmental conditions are being targeted and the methods used to change them. There also may be challenges when distinguishing between environmental change agents and EBI implementers, as their roles can sometimes overlap. For example, in the VSS intervention, the coalition was an implementer (e.g. helped develop infrastructure and organize intervention events) and also an environmental change agent by increasing access to physical activity opportunities in the community. The guided prompts and examples provided in the EBI Mapping resources can help to distinguish these EBI audiences, but it is possible for some EBI components to remain unclear even when all the materials are available. Another common challenge to understanding intervention components is inconsistent terms. As part of the EBI Mapping materials, terms are clearly defined for users to help improve consistency. Some intervention developers, however, may not clearly distinguish between or define their terms (e.g. classifying change methods as theoretical determinants). As a result, a planner using EBI Mapping needs to be aware of the potential differences between the EBI Mapping approach and the way existing interventions are described. 

## Conclusions

Despite the availability of numerous EBIs, planners often lack the information necessary to determine their appropriateness for their target population and setting. EBI Mapping represents a systematic approach to gain a comprehensive understanding of an existing EBI and inform decision making. More specifically, EBI Mapping can help planners when making decisions about EBI selection and adaptation. Using existing EBIs can save resources and maximize the benefits of interventions known to be effective. Overall, EBI Mapping can serve as a valuable tool for EBI selection, adaptation, and scale-up.

## Data Availability

All data generated or analyzed during this study are included in this published article.

## Abbreviations

EBI: Evidence-based interventions
VSS: VERB Summer Scorecard
Center TRT: Center for Training and Research Translation
FTE: Full-time equivalent
